# *Trypanosoma cruzi*, the Causal Agent of Chagas Disease: Boundaries between Wild and Domestic Cycles in Venezuela

**DOI:** 10.3389/fpubh.2014.00259

**Published:** 2014-11-28

**Authors:** Leidi Herrera

**Affiliations:** ^1^Laboratory of Parasite and Vector Biology, Institute of Tropical Zoology and Ecology, Science Faculty, Central University of Venezuela, Caracas, Venezuela

**Keywords:** *Trypanosoma cruzi*, domestic cycle, wild cycle, Chagas disease, Venezuela

## Abstract

*Trypanosoma cruzi* the etiological agent of American Trypanosomiasis or Chagas disease (ChD) is transmitted by triatomines vectors between mammals including man. *T. cruzi* has existed for *circa* 150 Ma in the Americas and nearly 10 million people are currently infected. The overlap between wild and domestic ecotopes where *T. cruzi* circulates is increasing. Host–parasite interactions have been determined by infection patterns in these cycles, all under natural or laboratorial conditions. This mini-review describes specific parasite niches, such as plant communities or biological corridors between domestic and wild landscapes, in order to help identify risk factors for ChD and define the boundaries between wild and domestic transmission cycles, with an emphasis on research undertaken in Venezuela.

## Introduction

**Figure 1:**
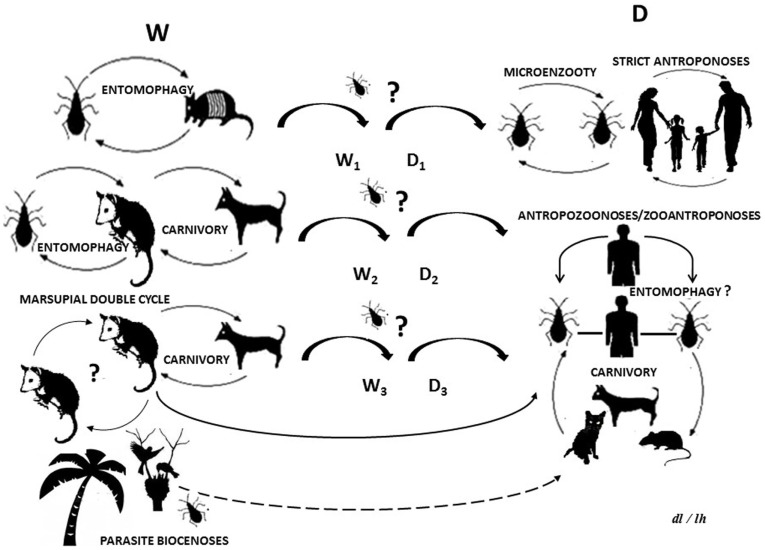
**Life cycle of *Trypanosoma cruzi*: (W) wild cycle with enzootic parasite circulation between wild and synanthropic mammals and other biotic components**. (D) Domestic cycle with parasites circulating as a zooanthroponosis, anthropozoonosis, strict anthroponosis, or micro-enzooty. (W_n_) Wild sub-cycles (D_n_) domestic sub-cycles. Question marks indicate uncertainty as regards parasite circulation patterns or processes. The dotted line indicates a hypothetical route.

Parasites and their hosts form part of trophic webs and may be considered bioindicators of climate changes and anthropogenic impacts (1). American trypanosomiasis (AT) or Chagas disease (ChD) is a complex parasitosis caused by *Trypanosoma cruzi* (Kinetoplastida, Trypanosomatidae), which can be dispersed by enzootic or anthroponotic routes in trophic webs, which involve several mammals groups including human beings (Figure 1). So, this parasite affects currently, until 10 million people and as such can be considered a re-emerging public health problem especially in Venezuela (2, 3).

The *T. cruzi* life cycle begins when vectors (Hemiptera, Reduviidae, Triatominae) expel feces or urine with infective metacyclic trypomastigotes which then come into contact with mammals via intact mucous or skin abrasions. The trypomastigotes pass into the bloodstream and invade a wide range of tissues where they differentiate into amastigotes, epimastigote, and trypomastigotes once again. The latter are re-released into the bloodstream and can be imbibed by another or the same vector, which pass into the intestine and transform, once more, into metacyclic, performing a vectorial transmission (3).

Recent outbreaks of oral transmission in Brazil and other Latin American countries, including Venezuela, emphasize the importance of this alternative route in enzootic and zoonotic cycles (4).

*Trypanosoma cruzi* has been grouped into six discrete typing units (DTU): *T. cruzi* I (TcI) to *T. cruzi* VI (TcVI). The TcI–TcVI classification is, however, a relatively recent nomenclature and the associations of the different genotypes with particular hosts, ecotopes, or transmission cycles remains under debate (5).

*Trypanosoma cruzi* has existed in the Americas for *circa* 150 Ma and has been in contact with Amerindians for 15,000 years. The genome of this parasite in mummies from the American Pacific (7,500 BC to 1,500 AC) indicates a pre-Columbian origin thus breaking the myth of its establishment as a product of recent colonialism (6).

The host–parasite associations and risk factors associated with ChD is being described in recent studies of specific niches such as mammal caves, plant communities, and biological corridors between domestic and wild ecotopes, in order to widen the understanding of the boundaries between wild and domestic *T. cruzi* cycles in Venezuela.

## Vector–Parasite: Patterns of Wild and Domestic *Trypanosoma cruzi* Cycles

Triatomines are eclectic in their ecological niches: they are found from 42°N to 46°S and between 400 and 1200 m.a.s.l. (7). A total of 140 species are grouped into 8 tribes and 15 genera. *Triatoma maculata*, *Rhodnius prolixus*, and *Panstrongylus geniculatus* are the most important in Venezuela by their frequency of infection by *T. cruzi* and their association with domestic and peridomestic ecotopes in economically depressed rural areas (8).

*Rhodnius prolixus* in Venezuela, is predominantly intradomiciliary with a high-reproduction rates, a voracious blood intake and fast defecation time, all of which are attributes of a primary vector. In the wild, this triatomine is predominantly found in palms with synanthropic vertebrates providing the blood source (9, 10). *T. maculata* is found in palms, dry trees, wooden fences, and bird nests near human dwellings. Its domiciliation, in function to phenotypic and genotypic discrimination according to its ecotopes, guarantee the previous consideration about its presence a risk factor for parasite transmission in Brazil, Colombia, and Venezuela (11–13).

Coconut palms (*Coccus nucifera*) is a suitable triatominae ecotope in peridomicile environments in north-eastern Venezuela, as was corroborated by the presence of 242 *R. prolixus* and 144 *T. maculata* adults in 14 coconut palms just 5 m away from human habitations. PCR amplification of the D7 divergent domain of the 24S rRNA genes; the non-transcribed spacer of mini-exon genes and the size-variable domain of the 18S rRNA genes confirmed that 98% of the *R. prolixus* and 70% of the *T. maculata* individuals were infected by *T. cruzi*–TcI. Exploration of coconut and its derivatives in industry and ethno-botany could pose a risk by exposing human beings to contaminated triatomines fluids (14).

Other triatomines vector species can acts as boundaries between wild and domestic environments. Examples are *Eratyrus mucronatus* and *Panstrongylus rufotuberculatus* found in palms, tree holes, and the dens of animal reservoirs, in Bolivia, Colombia, and Venezuela (15, 16). *P. rufotuberculatus* is a widely dispersed triatomine; in Venezuela could be monitored in peridomiciles in Anzoátegui state when wildlife fauna was affected or natural enemies were altered. Inoculation of the intestinal content of these insects in murine model, shown invasion of chondral tissue, brain, and kidney, revealing novel clinical aspects to be considered in relation to ChD (16).

Other triatomine species is *P. geniculatus*, which has been associated to infection of rodents and marsupials in rural or domestic ecotopes. The loss of its natural niches has also promoted its avid penetration in human dwellings. This is particularly worrying as this insect has been imputed as parasite font in cases of oral transmission of ChD in Caracas, and other cities in Venezuela. The modification of reservoir niches by climate change or the human exploitation of landscapes favors its peridomestic and domestic colonization (8, 17, 18).

The recent report of K-DNA and satellite DNA of *T. cruzi* in the intestines of *P. geniculatus* from sites along the Orinoco River, near Amerindian settlements, was associated with records of this species in neighboring countries, which could constitute evidence of biological corridors of the parasite with potential impacts on indigenous populations (19).

## Reservoir–Parasite: The Interaction of Wild Mammals, Human Beings, and Domestic Animals

Up until now, 180 species have been identified as reservoirs included in Artiodactyla, Carnivora, Cingulata, Chiroptera, Didelphimorphia, Lagomorpha, Perissodactyla, Pilosa, Primates (including man), and Rodentia orders (18).

*Trypanosoma cruzi* is considered as euryxenic according to the range of reservoirs it inhabits and eurytopic as regards the different organs it infects. Alternative transmission routes have also been reported in order to fluctuations in reservoir subpopulations, which could explain the plasticity of this zoonosis and urban outbreaks. The parasite may be orally transmitted via ingestion of infected triatomines, contaminated food, blood, or viscera from reservoirs (18, 20).

Studies of the distribution patterns of *T. cruzi* genotypes should consider ecological peculiarities since that genetic diversity has on the outcome of zoonosis or human disease (20). *T cruzi* Z3 in the southern Amazon (*Trichomys* rodent–*T. cruzi* complex in Brazil) and *T. cruzi* TcIII in the northern Amazon (*Dasypus*–*T. cruzi* complex in Venezuela) provide instances of the expansion of these wild genotypes into urban cycles (21, 22).

Particularly, *Dasypus novemcinctus* form part of an ancient enzootic *T. cruzi* cycle in the touristically important north-eastern region of Venezuela. These mammals act as TcIII reservoirs, as has been shown by the PCR amplification of the intergenic region of HSP60 genes for *T. cruzi* and the restriction digest of PCR products by *Eco*RV. The interaction of *Dasypus novemcinctus* with human beings, domestic animals, and peridomestic triatomines in this region, may be an important risk factor (22).

The ubiquity of *T. cruzi* in mammal reservoirs and its effect on host fitness represents an element that has been scarcely studied. Parasite isolates from *D. marsupialis*, *R. prolixus*, and *T. maculata* from rural and urban areas of Venezuela have yielded 10^5^ flagellates/ml of blood in mice models, producing 80% mortality with neurological symptoms such as ataxia, paralysis, and sphincter relaxation. Alterations as meningo-encephalitis, edema of the neuropil and parasitism near vascular system could facilitate the hematological dispersion of the parasites. These neurological disorders could alter the behavior of mammals toward predators thus modifying parasite transmission in trophic web (23, 24).

## Conclusion

Parasitism implicates energy movement among organisms, affecting the interactions and robustness of some trophic webs. *T. cruzi* is a clonal parasite with wild and domestic cycles, some author has proposed that vector–mammal interaction and saturation vector feeding rates, depend on mammal density when the vector/mammal ratio is low and vector density when this ratio is high (25). The number of infected mammals is conditioned by their relative abundance, which thus influences their availability as a blood source for triatomines.

New incursion of some vectors or mammals reservoirs species in *T. cruzi* life cycle, is important in the epidemiology of AT and ChD. The potential trophic web can include ingestion of insects, contaminated food, or host carnivorous behavior, which could be the primary route for *T. cruzi* transmission in some wild cycles (Figure 1). Synanthropic mammals and vectors are not excluded from this, thus, providing a way by which the wild and domestic cycles could be crossed (26–28).

## Conflict of Interest Statement

The author declares that the research was conducted in the absence of any commercial or financial relationships that could be construed as a potential conflict of interest. The Guest Associate Editor Juan-Carlos Navarro declares that, despite being affiliated to the same institution as the author Leidi Herrera, the review process was handled objectively and no conflict of interest exists.

## References

[1] Lafferty KD Hechinger RF Shaw JC Whitney KL Kuris AM Food webs and parasites in a salt marsh ecosystem. In: Collinge R, editor. Disease Ecology: Community Structure and Pathogen Dynamics. Oxford: Oxford University Press (2006). p. 119–34.

[2] WHO (World Health Organization). Chagas Disease (American Trypanosomiasis). FactSheet No 340. [Documento en línea], [Consulta: Mayo 2014] (2014). Available from: http://www.who.int/mediacentre/factsheets/fs340/en/index.html

[3] Pinto Dias JC Epidemiologia. In: Brener Z Andrade Z Barral-Netto M, editors. Trypanosoma cruzi e doença de Chagas. Rio de Janeiro: Guanabara-Koogan Press (2000). p. 48–74.

[4] Alarcón de Noya B Díaz-Bello Z Colmenares C Ruiz-Guevara R Mauriello L Zavala-Jaspe R Large urban outbreak of orally acquired acute Chagas disease at a school in Caracas, Venezuela. J Infect Dis (2010) 201:1308–15. 10.1086/651608 20307205

[5] Zingales B Andrade SG Briones MR Campbell DA Chiari E Fernandes O A new consensus for *Trypanosoma cruzi* intraspecific nomenclature: second revision meeting recommends TcI to TcVI. Mem Inst Oswaldo Cruz (2009) 104:1051–4. 10.1590/S0074-02762009000700021 20027478

[6] Aufderheide AC Salo W Madden M Streitz J Buikstra J Guhl F A 9,000-years record of Chagas disease. Proc Natl Acad Sci U S A (2004) 101:2034–9. 10.1073/pnas.0307312101 14766963 PMC357047

[7] Carcavallo RU Galíndez I Jurberg J Lent H Dos vectores da doença de Chagas nas Américas. In: Cruz F, editor. Atlas of Chagas Disease Vectors in the Americas. (Vol. III), Rio de Janeiro: Fiocruz (1999). p. 747–890.

[8] Reyes-Lugo M *Panstrongylus geniculatus* Latreille1811 (Hemiptera: Reduviidae: Triatominae), vector de la enfermedad de Chagas en el ambiente domiciliario del centro-norte de Venezuela. Rev Biomed (2009) 20:180–205.

[9] Teixeira AR Monteiro PS Rebelo JM Argañaraz ER Vieira D Lauria-Pires L Emerging Chagas disease: Trophic network and cycle of transmission of *Trypanosoma cruzi* from palm trees in the Amazon. Emerg Infect Dis (2001) 1:100–12. 10.3201/eid0701.070100 11266300 PMC2631687

[10] Longa A Scorza JV Migración de *Rhodnius robustus* (Hemiptera, Triatominae) desde *Acrocomia aculeate* (Palmae) hacia domicilios rurales en Venezuela. Bol Mal Sal Amb (2007) 47:213–20.

[11] Cantillo-Barraza O Gómez-Palacio A Salazar D Mejía-Jaramillo A Calle J Triana O. Distribution and ecoepidemiology of the triatomine fauna (Hemiptera: Reduviidae) in Margarita Island, Bolívar, Colombia. Biomédica (2010) 30:382–9. 21713340

[12] Luitgards-Moura JF Vargas AB Almeida CE Magno-Esperança G Agapito-Souza R Folly-Ramos E A *Triatoma maculata* (Hemiptera, Reduviidae, Triatominae) population from Roraima, Amazon region, Brazil, has some bionomic characteristics of a potential Chagas disease vector. Rev Inst Med Trop São Paulo (2005) 47:131–7. 10.1590/S0036-46652005000300003 16021285

[13] García-Alzate R Lozano-Arias D Reyes-Lugo RM Morocoima A Herrera L Mendoza-León A. *Triatoma maculata*, the vector of *Trypanosoma cruzi*, in Venezuela. Phenotypic and genotypic variability as potential indicator of vector displacement into the domestic habitat. Front Public Health (2014) 2:170. 10.3389/fpubh.2014.00170 25325053 PMC4179684

[14] Morocoima A Chique J Zavala-Jaspe R Díaz-Bello Z Ferrer E Urdaneta-Morales S Commercial coconut palm as an ecotope of Chagas disease vectors in north-eastern Venezuela. J Vector Borne Dis (2010) 47:76–88. 20539044

[15] Morocoima A Chique J Herrera L Urdaneta-Morales S *Eratyrus mucronatus* (Stal, 1859) (Hemiptera, Reduviidae, Triatominae): primer registro para el estado Anzoátegui (Venezuela). Bol Mal Sal Amb (2012) 50:307–10.

[16] Morocoima A Coriano H Navas C De Sousa L Ferrer E Herrera L *Panstrongylus rufotuberculatus* (Hemiptera, Reduviidae, Triatominae) infectado con *Trypanosoma cruzi* en el estado Anzoátegui (Venezuela). Bol Mal Sal Amb (2012) LII:135–8.

[17] Carrasco H Torrellas A García C Segovia M Feliciangeli D Risk of *Trypanosoma cruzi* (Kinetoplastida: Trypanosomatidae) transmission by *Panstrongylus geniculatus* (Hemíptera: Reduviidae) in Caracas (Metropolitan District) and neighbouring states, Venezuela. Int J Parasitol (2005) 35:1379–84 10.1016/j.ijpara.2005.05.003 16019006

[18] Herrera L Una revisión sobre reservorios de *Trypanosoma* (*Schizotrypanum*) *cruzi* (Chagas, 1909), agente etiológico de la Enfermedad de Chagas. Bol Mal Sal Amb (2010) L:1–13.

[19] Noya-Alarcón O Botto C Cortez J Ferrer E Viettri M Herrera L Primer registro de *Panstrongylus geniculatus* (Latreille, 1811) en los municipios Alto Orinoco y Atures, Estado Amazonas, Venezuela. Bol Mal Sal Amb (2011) LI:81–5.

[20] Carrasco HJ Segovia M Llewellyn MS Morocoima A Urdaneta-Morales S Martínez C Geographical distribution of *Trypanosoma cruzi* genotypes in Venezuela. PLoS Negl Trop Dis (2012) 6:e1707. 10.1371/journal.pntd.0001707 22745843 PMC3383755

[21] Herrera L D’Andrea PS Xavier SCC Mangia RH Fernandes O Jansen AM. *Trypanosoma cruzi* in wild mammals of the National Park “Serra da Capivara”, and its surroundings (Piauí, Brazil), endemic for Chagas disease. Trans R Soc Trop Med Hyg (2005) 99:379–88. 10.1016/j.trstmh.2004.07.006 15780345

[22] Morocoima A Carrasco HJ Boadas J Chique JD Herrera L Urdaneta-Morales S. *Trypanosoma cruzi* III from armadillos (*Dasypus novemcinctus novemcinctus*) from Northeastern Venezuela and its biological behavior in murine model. Risk of emergency of Chagas disease. Exp Parasitol (2012) 132:341–7. 10.1016/j.exppara.2012.08.008 22902748

[23] Morocoima A Socorro G Ávila R Hernández A Merchán S Ortiz D *Trypanosoma cruzi*: experimental parasitism in the central nervous system of albino mice. Parasitol Res (2012) 111:2099–107. 10.1007/s00436-012-3057-9 22868891

[24] Teixeira ARL Hecht MM Guimaro MC Sousa AO Nitz N. Pathogenesis of Chagas’ disease: parasite persistence and autoimmunity. Clin Microbiol Rev (2011) 24:592–630. 10.1128/CMR.00063-10 21734249 PMC3131057

[25] Roellig DM Ellis AE Yabsley MJ. Oral transmission of *Trypanosoma cruzi* with opposing evidence for the theory of carnivory. J Parasitol (2009) 95:360–4. 10.1645/GE-1740.1 18763853 PMC2911628

[26] Kribs-Zaleta CM. Alternative transmission modes for *Trypanosoma cruzi*. Math Biosci Eng (2010) 7:661–76. 10.3934/mbe.2010.7.657 20578791

[27] Herrera HM Rocha FL Lisboa CV Rademaker V Mourão GM Jansen AM. Food web connections and the transmission cycles of *Trypanosoma cruzi* and *Trypanosoma evansi* (Kinetoplastida, Trypanosomatidae) in the Pantanal Region, Brazil. Trans R Soc Trop Med Hyg (2011) 7:380–7. 10.1016/j.trstmh.2011.04.008 21600622

[28] Kribs-Zaleta CM. Estimating contact process saturation in sylvatic transmission of *Trypanosoma cruzi* in the United States. PLoS Negl Trop Dis (2010) 4(e):656. 10.1371/journal.pntd.0000656 20436914 PMC2860507

